# Rapid mechanical phenotyping of breast cancer cells based on stochastic intracellular fluctuations

**DOI:** 10.1016/j.isci.2024.110960

**Published:** 2024-10-04

**Authors:** Álvaro Cano, Marina L. Yubero, Carmen Millá, Verónica Puerto-Belda, Jose J. Ruz, Priscila M. Kosaka, Montserrat Calleja, Marcos Malumbres, Javier Tamayo

**Affiliations:** 1 Bionanomechanics Lab, Instituto de Micro y Nanotecnología, IMN-CNM (CSIC), Tres Cantos, Madrid, Spain; 2 Cancer Cell Cycle Group, Vall d’Hebron Institute of Oncology (VHIO), Barcelona, Spain

**Keywords:** Cancer systems biology, Cancer

## Abstract

Predicting the phenotypic impact of genetic variants and treatments is crucial in cancer genetics and precision oncology. Here, we have developed a noise decorrelation method that enables quantitative phase imaging (QPI) with the capability for label-free noninvasive mapping of intracellular dry mass fluctuations within the millisecond-to-second timescale regime, previously inaccessible due to temporal phase noise. Applied to breast cancer cells, this method revealed regions driven by thermal forces and regions of intense activity fueled by ATP hydrolysis. Intriguingly, as malignancy increases, the cells strategically expand these active regions to satisfy increasing energy demands. We propose parameters encapsulating key information about the spatiotemporal distribution of intracellular fluctuations, enabling precise phenotyping. This technique addresses the need for accurate, rapid functional screening methods in cancer medicine.

## Highlights

• Noise decorrelation method allows imaging of fast intracellular fluctuations by QPI
• Unexpectedly, thermal and ATP-driven fluctuations coexist in breast cancer cells
• ATP-driven regions within the cell expand with malignancy
• Spatiotemporal features of the fluctuations allow rapid cell phenotyping

## Graphical abstract

**Figure:**
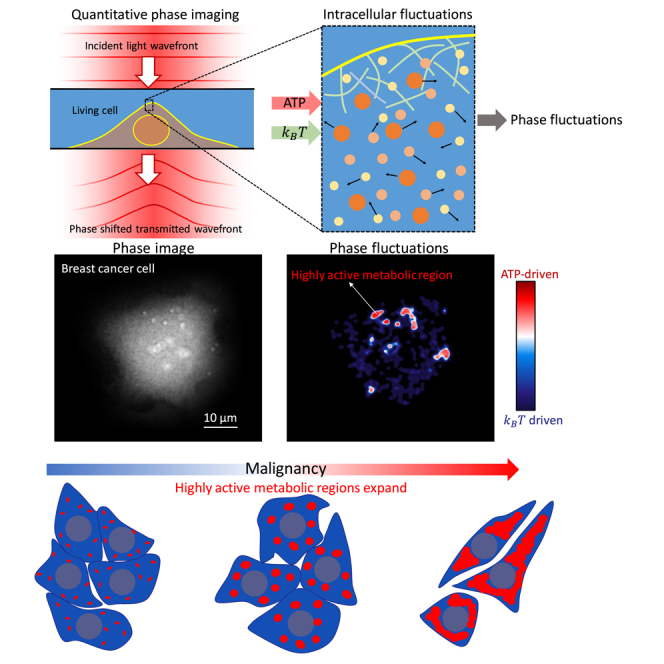

## Introduction

Genome sequencing studies have unveiled a vast array of genetic alterations in a broad spectrum of cancers. Yet, the challenge of predicting the impact of these genetic alterations in the cell phenotypes remains an unmet need in medical genetics.^1^^,^^2^^,^^3^ High-throughput optical screening methods have become indispensable in functional screening, serving as a key tool in identifying the relationship between intricate genetic modifications and disease progression, malignancy, and drug resistance.^3^^,^^4^ Optical techniques encompass the morphological analysis of cells to pinpoint unique characteristics linked to the state of the disease, fitness-based assays with a particular focus on growth rate to examine proliferation rate, and migration assays to investigate the metastatic potential.^4^^,^^5^^,^^6^ Whereas these techniques offer insights on the functional impact of cancer genetic variants, these still come with limitations in reproducibility, sensitivity, and specificity. Optical microscopy suffers from poor optical contrast, providing only qualitative information. Fluorescence microscopy offers higher specificity due to its capability for imaging fluorescence-labeled biomarkers; however, fluorescent dyes can introduce cytotoxicity effects and potential artifacts and are prone to photobleaching, which prevents long-term observation of cells.^7^^,^^8^ Therefore, there is an acute need for simple, cost-effective, and noninvasive methods that can accurately monitor in real time biophysical parameters linked to malignant transformation, tumor progression, metastasis, and therapy efficacy.^9^^,^^10^^,^^11^^,^^12^^,^^13^^,^^14^

In this context, quantitative phase imaging (QPI) emerges as a promising noninvasive and label-free approach for imaging cells. It combines elements of microscopy, holography, and light-scattering techniques to generate quantitative images of cell-induced phase delays with nanoscale sensitivity to morphology and dynamics.^15^^,^^16^^,^^17^^,^^18^^,^^19^^,^^20^ QPI has demonstrated high sensitivity to detect subtle structural changes associated with malignant transformation, phenotype, progression, and treatment.^20^^,^^21^^,^^22^^,^^23^^,^^24^^,^^25^^,^^26^ One of the most impactful attributes of QPI is the unique ability for providing quantitative maps of the dry mass, the mass of the non-aqueous content of the cell, namely proteins, nucleic acids, carbohydrates, and lipids.^27^^,^^28^^,^^29^^,^^30^ The dry mass can be regarded as the most precise indicator of the biosynthetic and degradative processes within a cell. The QPI capability for real-time monitoring of cell dry mass over extended periods is a distinctive attribute for cancer functional screening and precision oncology, where targeting uncontrolled proliferation is a primary focus of many therapies and genetic variants.^31^^,^^32^ However, one of the main limitations of these assays is the lengthy analysis time required, ranging from 20 h to several days.

Little attention has been given to another essential cancer hallmark: deregulated cell energetics, which confers cells with a variety of metabolic strategies for deregulated proliferation and metastasis.^33^^,^^34^ Studies of the motion of intracellular fluorescence micro-nanoparticle probes have suggested that stochastic intracellular fluctuations at short time scales, from milliseconds to seconds, encode metabolic indicators for phenotyping cell malignancy.^35^^,^^36^^,^^37^ Despite the attractiveness of this approach, the experimental evidence is not solid, due to the limitations associated with fluorescence particle tracers—such as phototoxicity, low throughput, and localized information—which may not reflect the remarkable spatial heterogeneity within cells.^8^^,^^38^^,^^39^^,^^40^ QPI has been applied to the measurement of slow or large mass redistribution processes,^41^^,^^42^^,^^43^^,^^44^^,^^45^^,^^46^ but visualization of stochastic fluctuations at shorter temporal scales has been out of reach due to temporal phase noise, which ultimately limits QPI sensitivity.^18^^,^^19^^,^^20^^,^^47^

We report the development of a noise decorrelation algorithm and image processing methods for QPI, which enable the visualization of rapid intracellular fluctuations with unmatched sensitivity. This method is applied to breast cancer cells of varying malignancy degrees. Our analysis of the spatiotemporal patterns in the intracellular noise reveals reliable indicators of the cells’ metabolic state and malignancy. Furthermore, our findings disclose a previously unobserved coexistence of passive and highly active regions within the cells. Notably, we observe that the cells strategically expand and increase the number of active regions to meet the escalating energy demands associated with increased malignancy.

## Results

### QPI and phase noise decorrelation algorithm

**Figure 1:**
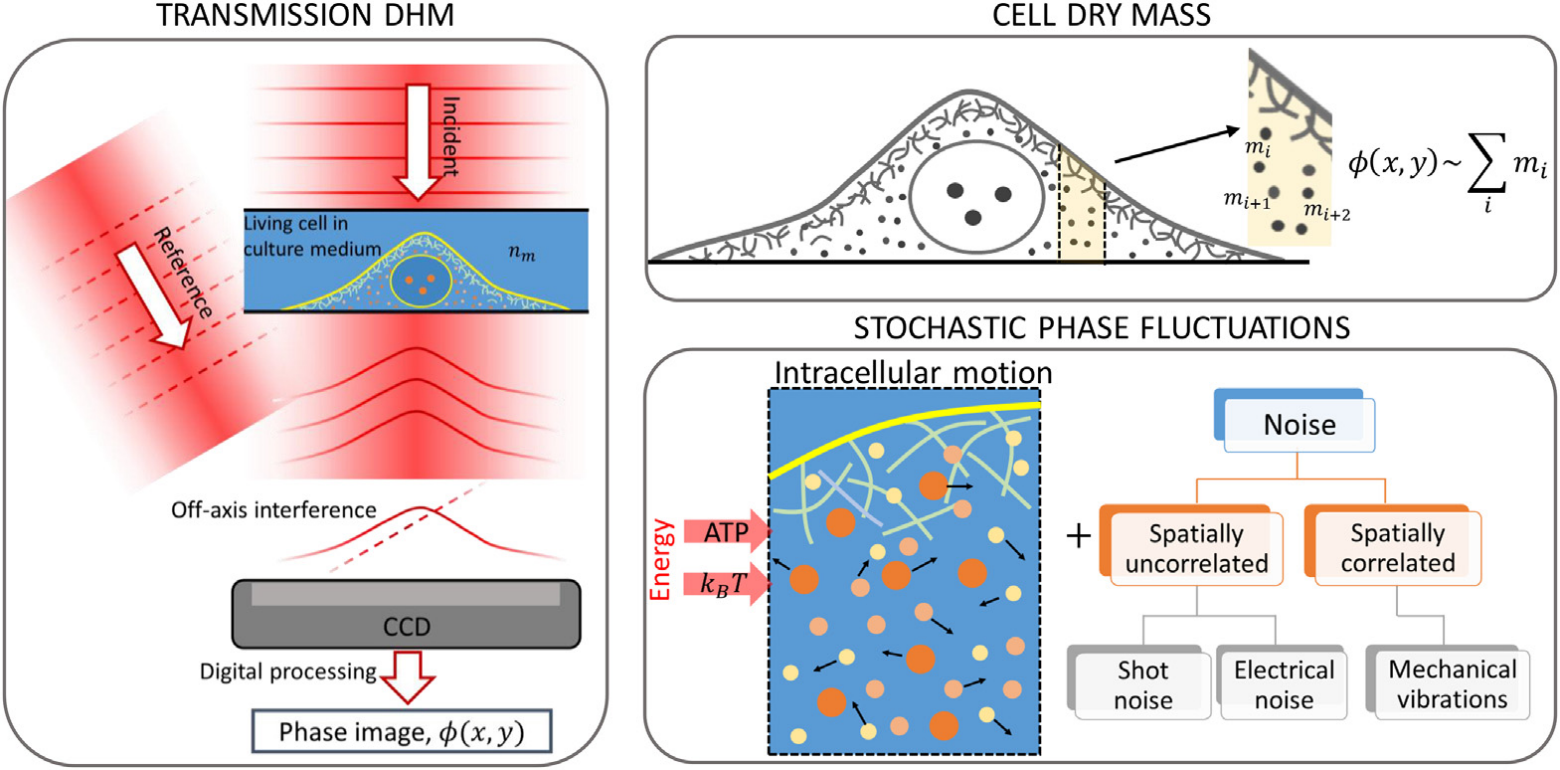
Schematics of quantitative phase digital holographic microscopy for imaging intracellular mass fluctuations in living cells QP-DHM measures the amplitude and phase of the light wavefront transmitted through the cell by using off-axis Mach-Zehnder interferometer. Phase variations, ϕ(x,y), arise from the mass of the non-aqueous components of the cell (proteins, nucleic acids, carbohydrates, and lipids), referred to as dry mass. Dry mass fluctuations due to intracellular trafficking give rise to small phase fluctuations. These fluctuations are driven by thermal energy and ATP hydrolysis. Phase noise obscures intracellular dry mass fluctuations. This noise is categorized into spatially uncorrelated noise, such as optical and electrical noise and spatially correlated noise, namely arising from mechanical vibrations that are the dominant source of noise.

We use a transmission quantitative phase digital holographic microscopy (QP-DHM) for imaging the breast epithelial cells in their optimal culture conditions (Figure 1). This technique measures the amplitude and phase of the light wavefront transmitted through the cell by using an off-axis Mach-Zehnder interferometer.^15^^,^^16^^,^^17^^,^^18^^,^^19^^,^^20^ The phase shift can be written as

(Equation 1) $$\phi =\frac{2\pi }{\lambda }\int_{0}^{h}\left(n_{c}\left(z\right)-n_{m}\right)dz$$

where λ is the light wavelength, h, the cell’s height, and $n_{c}$ and $n_{m}$, the refractive index of the cell and the culture medium, respectively. The equivalent variation of the optical path distance (OPD) is given by $\frac{\lambda }{2\pi }\phi \left(x,y\right)$. In homogeneous solutions of biological molecules, the refractive index increment, $n_{c}\left(z\right)-n_{m}$, is proportional to the concentration. Interestingly, the proportionality constant referred to as the specific refractive index increment, α, falls within a very narrow distribution for most of the intracellular constituents, including proteins, nucleic acids, carbohydrates, and lipids. By assuming a constant value of *α* = 1.9 × 10^−4^ m^3^/kg, the dry mass surface density, *σ*, of the cell can be obtained within an error of 3% from the OPD by using^27^^,^^28^^,^^29^^,^^30^^,^^48^^,^^49^

(Equation 2) $$\sigma \left(x,y\right)=\frac{OPD\left(x,y\right)}{\alpha }$$

Intracellular mass fluctuations are primarily governed by the stochastic movements of proteins, which are driven by inherent thermal forces and the cumulative effect of multiple motors, resulting in an incoherent background of fluctuating forces.^35^^,^^36^^,^^37^ The first category of motion is termed “passive fluctuations,” while the latter is known as “active fluctuations,” and it relies on adenosine triphosphate (ATP) hydrolysis. Identifying the phase signal associated with intracellular mass fluctuations presents a significant challenge, primarily due to the temporal phase stability, a critical constraint in QPI. This issue is further exacerbated at shorter time scales (less than 100 s), such as those examined in this study. Phase noise primarily arises from the processes of photon counting and the conversion of these counts into electrical signals, and mechanical vibrations that cause fluctuations in the optical path difference between the reference and object beams.^18^^,^^19^^,^^20^^,^^47^ The latter source of noise largely dominates even after mechanical isolation of the microscope by using passive and active components. Common path QPI configurations, in which both the object and reference beams traverse the same optical path, have been suggested as a solution to this issue.^50^^,^^51^ While this approach can indeed mitigate phase noise, it introduces other constraints related to spatial and temporal resolution.^18^ Furthermore, common path phase noise rejection only suppresses noise from the fluctuations of the optical elements involved the entire optical path, but noise originating from mechanical vibrations of the sample remains unaffected. When observing living cells in culture conditions, dominant phase noise stems from mechanical vibrations impacting the perfusion chamber housing the cells. A key feature of this source of phase noise is that it is spatially correlated. Background vibrations in the building are transmitted through mechanical waves to the sample with a spatial distribution determined by the geometry of the instruments and used materials. We exploit the spatial coherence of these fluctuations to develop a noise-suppression method based on signal decorrelation.

**Figure 2:**
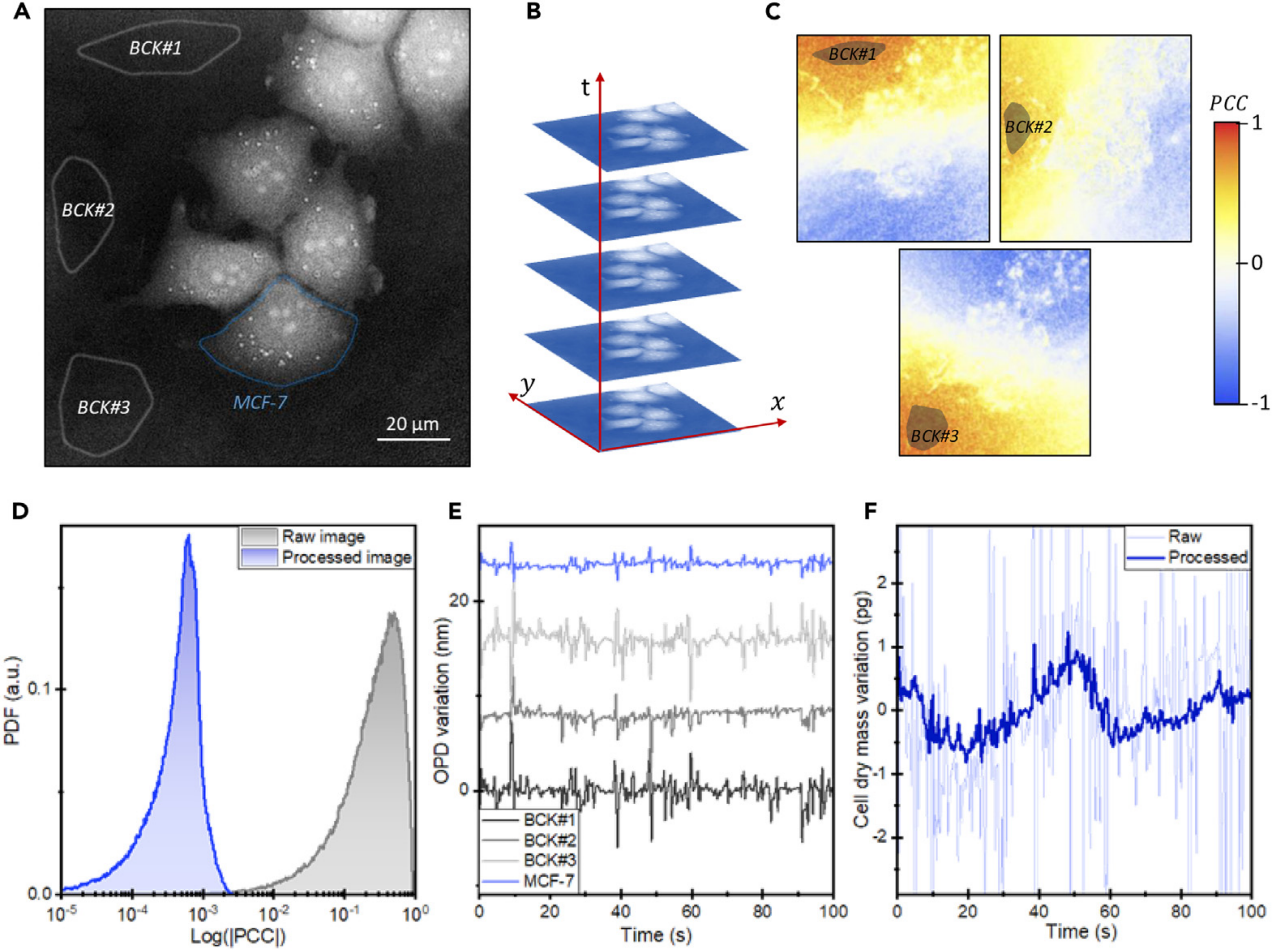
Spatial decorrelation of the phase noise (A) An OPD image of MCF-7 cells on glass. The cell temporal series are isolated from the background by creating masks for each cell and for the background (BCK#) at three distinct regions far from the cells. (B) OPD images of the sample were acquired at a rate of 20 frames per second for a duration of 100 s. (C) Images of the Pearson correlation coefficient (PCC) of the OPD temporal series of image pixels with the spatially averaged OPD at the three background regions. (D) Mean probability density function (PDF) of the logarithm of the absolute value of the PCCs before (raw) and after (processed) application of the decorrelation algorithm. (E) The mean OPD signal at the marked MCF-7 cell in (A) and at the three background regions prior to the application of the decorrelation algorithm. The offset of each OPD trajectory has been adjusted to facilitate comparison. (F) The estimated variation in the cell dry mass of the marked MCF-7 cell before (raw) and after (processed) the application of the decorrelation algorithm.

Our decorrelation method is illustrated with an experiment, where MCF-7 breast cancer cells were imaged at 20 frames per second over 100 s (Figures 2A and 2B). Here, we describe the key features of the method, with the full mathematical description provided in Methods S1. The method starts by designing masks for each cell and selecting background regions of the sample that are well separated from cells. The spatially averaged OPD at these background regions serves as references for our decorrelation method. The spatially correlated noise is evaluated by calculating the Pearson correlation coefficient (PCC) between the time series of the image pixels and the background references (Figure 2C). The resulting PCC maps demonstrate that spatially correlated phase noise was the predominant source of phase fluctuation. The dependency of the PCC maps on the used reference reflects the complexity of the spatial patterns associated with the spatially correlated noise.

The underlying model of the decorrelation technique is founded on the assumption that the spatially correlated noise can be approximated by a linear combination of the reference OPDs. The accuracy of the approximation relies on the judicious selection of the background regions to capture the spatial coherence of the noise, as well as the number of background regions. In our experiments, we found that optimal decorrelation was achieved by selecting three or four well-distributed background regions within the sample. Under this approximation, the OPD fluctuation at each position of the cell can be expressed as

(Equation 3) $$s\left(x,y,t\right)=u\left(x,y,t\right)+\sum_{i=1}^{N}\alpha_{i}\left(x,y\right)r_{i}\left(t\right)$$

where u is the OPD arising from the intracellular fluctuations, the second term represents the influences of the fluctuations of the reference signals, $r_{i}$, accounted by the coefficients $\alpha_{i}$, and N is the number of reference signals. Provided that the intracellular stochastic fluctuations and the spatially correlated noise are uncorrelated in time, the covariance of the OPD signal with the reference OPD signals is given by

(Equation 4) $$\left\langle s\left(x,y,t\right)r_{j}\left(t\right)\right\rangle ≅\sum_{i=1}^{N}\alpha_{i}\left(x,y\right)\left\langle r_{i}\left(t\right)\;r_{j}\left(t\right)\right\rangle$$

The system of Equation 4 can be computed for each pixel of our images, with the unknowns given by coefficients $\alpha_{i}$. Resolution of this problem leads to an accurate estimation of the OPD that arises from intracellular fluctuations (see Methods S1 and Figure S1). The decorrelation algorithm effectively reduces the PCCs of the image by approximately three orders of magnitude (Figure 2D). The efficacy of this method is illustrated by analyzing the mean OPD signal of an MCF-7 cell relative to the background reference signals (Figure 2E). Temporal phase noise makes indistinguishable the mean OPD variations of the cell from those of the selected background regions. However, after application of the decorrelation algorithm, the intrinsic variations of the cell dry mass are detectable.

We examine the effect of the decorrelation method on the OPD temporal noise, one of the most important parameters that define the performance in QPI (see Methods S1 and Figure S1). The standard deviation of temporal fluctuations in background regions sets the OPD noise. While spatial and temporal averaging methods are typically proposed for noise reduction, our focus on fast intracellular fluctuations precludes the use of time averaging, to capture rapid fluctuations.^11^^,^^19^^,^^29^^,^^30^ We turn our attention to the effect of the spatial bandwidth. The standard deviation of the OPD per pixel (0.28 μm pixel size) at the background regions is 2.7 nm. Spatial averaging reduces this noise up to averaging area radius of about 1–1.2 μm (≈36 pixels). Beyond this point, the OPD noise reaches an asymptotic behavior of 1.1 nm for larger spatial averaging. The little reduction of the phase noise with spatial averaging is consistent with our results, indicating that spatially correlated temporal phase noise is dominant. This sets a minimum detectable variation of the dry mass of the cells (with spatial averaging of approximately 700 μm^2^, equivalent to 9,000 pixels) of about 4 pg, consistent with previous studies.^11^^,^^19^^,^^29^^,^^30^Upon application of the decorrelation algorithm, the phase noise in the background regions becomes white, benefiting from spatial averaging with the number of pixels as approximately $1/\sqrt{N}$. While OPD noise per pixel reduces by 20%, for features with 1 μm radius, the OPD noise reduces 3-fold from 1.2 nm to 0.4 nm. For cell integration areas, the OPD noise reduces further to 0.03 nm, setting the minimum detectable cell mass variation at about 0.1 pg, a 40-fold improvement. The achieved dry mass resolution surpasses those previously obtained with QPI methods (1–10 pg) without need of time averaging and other ultrasensitive techniques like microcantilever mass sensors (1–10 pg).^52^ It is worth to note that these devices cannot quantitatively measure the cell mass, as these measurements are highly dependent on cell mechanical properties.^13^ This places QPI at the level of the highest resolution achieved in cell mass measurements obtained with suspended microchannel resonators (0.05 pg). However, this technique is limited to cells in suspension and is not applicable to adherent cells.^53^^,^^54^^,^^55^

Following the application of the decorrelation algorithm, the OPDs at the cell and the references are treated as uncorrelated. We estimated the power of the intracellular fluctuations for each pixel within the cell by subtracting the OPD pixel noise power derived from the references (see STAR Methods and Figure S3).

### Indicators of intracellular fluctuations

**Figure 3:**
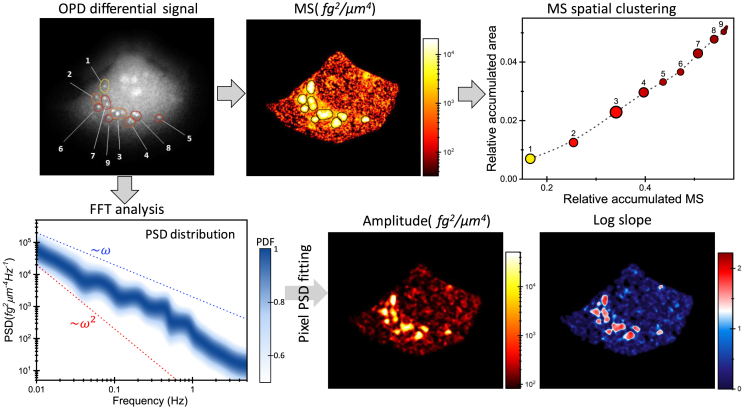
Process for analyzing the intracellular dry mass fluctuations illustrated with an MCF-7 cell (Top, left), OPD image of the MCF-7 cell. (Top, middle), image of the MS of the dry mass surface density over a 100 s interval. (Top, right), relative accumulated area vs. the relative accumulated MS of the regions of highest MS (hotspots). The hotspots are isolated by selecting the pixels that constitute 68.2% of the MS. The hotspots are numbered at the OPD image for their identification. The size of the symbols scales with the size of the isolated active regions, and the color intensity scales with the corresponding MS. (Bottom, left), probability density function of the power-spectral densities (PSDs) of the cell pixels. Most of the PSDs can be described by irregular variations around a linear baseline in the log-log scale. Each pixel PSD is fitted to a line in the log-log scale. (Bottom, middle) and (Bottom, right), images corresponding to the best-fitting parameters of the PSD, the power amplitude at 0.01 Hz and log slope, respectively.

In this study, we established a methodological pipeline to scrutinize the spatiotemporal distribution of intracellular dry mass fluctuations, as illustrated in an MCF-7 cell (Figure 3). First, we examined the spatial distribution of the fluctuations by calculating the mean square (MS) of each cell pixel over 100 s intervals. The resulting image revealed areas of very high activity, or “hotspots,” in close proximity to the nucleus, amid a dispersed background of lesser activity throughout the cell. To isolate these hotspots, the most active pixels that contribute to a fraction of the total MS of the cell were selected. In this work, this fraction was set to 0.682% (see STAR Methods). The method allowed for the isolation of clusters of connected pixels with very high activity for their subsequent morphological analysis. After that, it was shown that the MS hotspots corresponded with round structures in the OPD image, exhibiting a typical radius of 0.6–0.7 μm, that have previously been identified as mitochondria.^41^^,^^56^ Interestingly, just seven of these structures, which make up only 4% of the total cell area, account for 50% of the total metabolic activity of the cell (Figure 3). This illustrates, as corroborated later, how the intracellular activity in cells is highly localized.

A second class of analysis involves examining the distribution of the intracellular fluctuations in the frequency domain. A prevalent feature observed in the power-spectral densities (PSDs) at the cell pixels is an approximate linear dependence on frequency when plotted on a log-log scale, albeit with some superimposed irregular variations (Figure S3). To understand this behavior, we refer to stochastic models on the diffusion of intracellular particles (Methods S1 and Figure S4).^35^^,^^36^^,^^37^^,^^57^^,^^58^^,^^59^^,^^60^ These studies elucidate how the logarithmic slope of the PSDs enclose valuable insights into the nature of the fluctuations. For passive fluctuations, the PSD logarithmic slope is 1+β, where β is the power-law exponent that describes the complex stiffness of the viscoelastic cell interior that follows ${∼\left(i\omega \right)}^{\beta }$. In living cells, β typically falls within 0.1–0.2 for frequencies lower than ≈100 Hz.^35^^,^^60^ Conversely, the PSDs of active fluctuations display a logarithmic slope of 2(1+β) for frequencies $\omega \gg \frac{1}{\tau }$, where τ is the mean processivity time of the motors, typically 5–10 s. For low frequencies, $\omega \ll \frac{1}{\tau }$, the logarithmic slope is 2β. These studies demonstrate that the logarithmic slope serves as a reliable indicator of metabolic activity, with slopes near 1 signifying passive fluctuations (the system is near thermodynamic equilibrium) and slopes near 2 signifying active fluctuations (the system is out of equilibrium). In our method, each PSD at each pixel averages the fluctuations of 10^4^ to 10^5^ proteins, each experiencing different ATP-dependent forces and different viscoelasticity depending on its position, particularly along the z direction. Consequently, the logarithmic slopes generally vary in the range from 1 to 2, scaling with ATP-dependent activity. The resulting MCF-7 cell image of the logarithmic slope after fitting each pixel PSD to a linear model in the log-log scale shows that slopes near 2 are almost exclusively found in the previously identified MS hotspots, whereas the slope is near 1 in the rest of the cell (Figure 3). This behavior departs with respect to the paradigm logarithmic slopes near 1 for fluctuations in nonactive environments and near 2 for fluctuations in active environments, such as within a living cell.^35^^,^^60^ This further confirms the localized nature of the cell metabolic activity.

### Phenotyping breast cancer cells based on intracellular fluctuations

We study the intracellular dry mass fluctuations in three cell lines with increasing malignancy: MCF-10A, MCF-7, and MDA-MB-231, each representing different aspects of breast cancer biology. MCF-10A is a nontumorigenic human breast epithelial cell line, used as a control. MCF-7 cells are used as model for hormone-responsive breast cancers that are characterized by low metastatic potential, and MDA-MB-231 is a cell line model for triple-negative breast cancers that has high metastatic potential. The study is carried out in optimal culture conditions and under ATP depletion to inhibit motor activity and protein transport processes. The details regarding the number of independent experiments, number of cell analyzed, and the specific culture conditions are comprehensively outlined in the STAR Methods.

**Figure 4:**
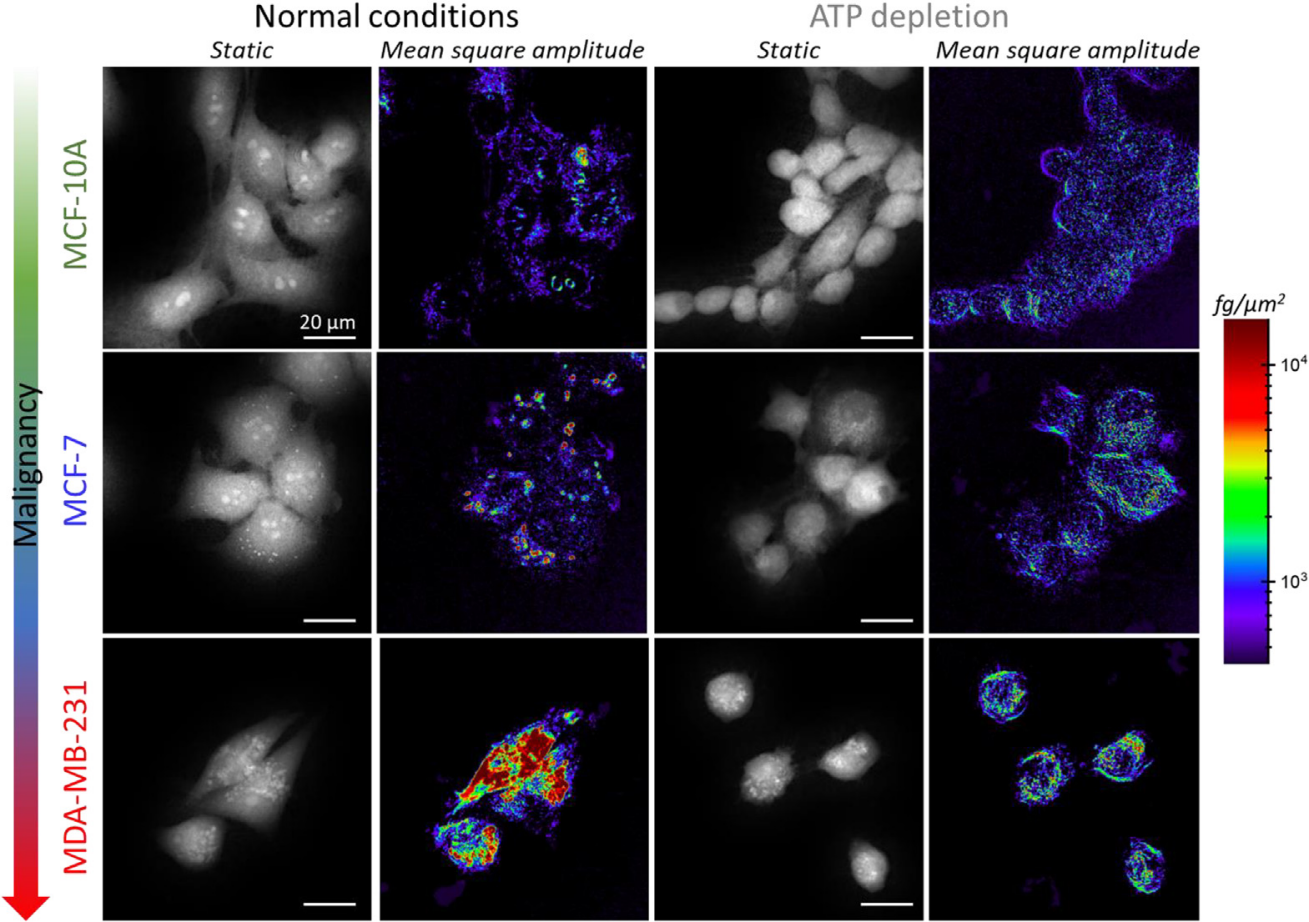
Representative images of the optical path distance and the mean square of the intracellular dry mass fluctuations for MCF-10A, MCF-7, and MDA-MB-231 cells These images were captured under optimal culture conditions and ATP depletion. All images share a common scale bar of 20 μm. The images depicting the mean square of the dry mass fluctuations were computed from the 100-s trajectory of each pixel, measured at a frequency of 20 Hz.

The morphological features exhibited by MCF-10A, MCF-7, and MDA-MB-231 cells were captured in OPD images, while the intracellular activity was imaged by computing the MS of the intracellular dry mass (Figure 4). Under optimal culture conditions, we found an increase of the dry mass fluctuations with the malignancy of the cells. In MCF-10A cells, the fluctuations were predominantly located on the cell periphery, with negligible activity in the nucleus, excluding the nucleoli. As previously described, MCF-7 cells displayed areas of high activity associated with the motion of round structures with a radius of 0.6–0.7 μm. MDA-MB-231 cells exhibited a marked increase in dry mass fluctuations, with extensive regions of high activity that also corresponded with regions of higher optical contrast. Further image processing revealed that this activity originated from the motion of the round structures previously identified in MCF-7 cells, albeit in greater quantities. The spatial patterns of the intracellular fluctuations underwent significant modifications under energy starvation. The fluctuations became more sparsely and uniformly distributed. Moreover, hotspots of activity were not observed in MCF-10 and MCF-7 cells. In MDA-MB-231 cells, although the fluctuations considerably decreased, regions of high activity persisted, suggesting that these structures remained metabolically active, albeit at a lower level than under nutrient-rich conditions.

**Figure 5:**
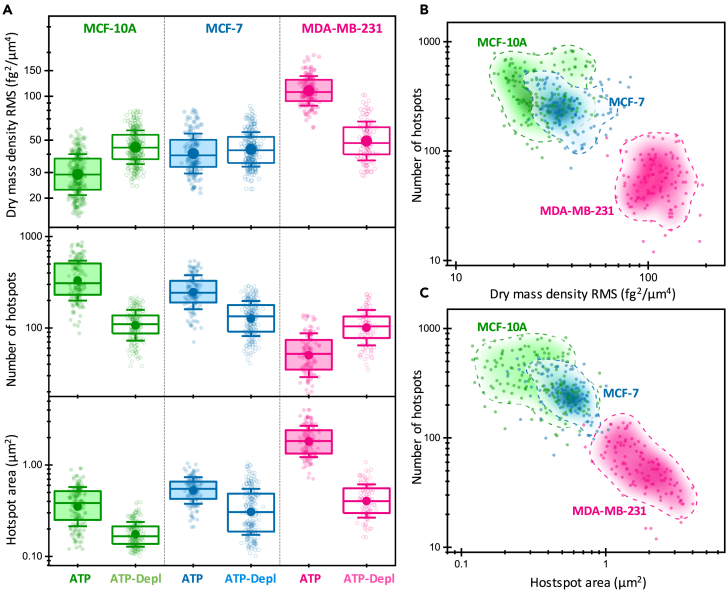
Analysis of the mean square fluctuations in the dry mass density over 100-s intervals for MCF-10, MCF-7, and MDA-MB-231 cells under both normal and ATP-depleted conditions (A) Boxplots of the root mean square (RMS) of the dry mass density (top), the number of the highly active regions (middle), and the average area of these regions (bottom). The symbols denote experimental values, the box indicates the (25%, 75%) quantiles, the large filled circle represents the mean, and the error bar shows the standard deviation. (B) Two-dimensional probability density function (PDF) showing the relationship between the number of highly active regions and RMSs of intracellular mass fluctuations, along with the experimental values. The dashed lines outline the region where the PDF reaches 20% of its maximum value. (C) Two-dimensional PDF depicting the correlation between the number and area of highly active regions, accompanied by the experimental values. The dashed lines demarcate the area where the PDF is 20% of its peak value.

**Figure 6:**
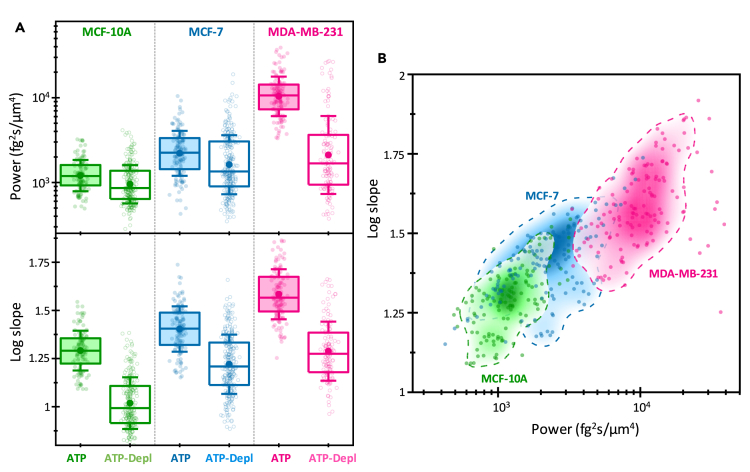
Analysis of the mean power of the dry mass density fluctuations at 0.01 Hz and the logarithmic slope of the power-spectral density obtained by linear fitting of the PSDs in log-log scale for MCF-10, MCF-7, and MDA-MB-231 cells under both normal and ATP-depleted conditions (A) Boxplots of the mean power (top) and log slope (bottom). The symbols denote experimental values, the box indicates the (25%, 75%) quantiles, the large filled circle represents the mean, and the error bar shows the standard deviation. (B) Two-dimensional probability density function (PDF) showing the relationship between the power amplitude and the log slope, along with the experimental values. The dashed lines outline the region where the PDF reaches 20% of its maximum value.

We now apply our previously established methodological pipeline for analyzing the spatiotemporal features of the intracellular fluctuations (see Figure 3 and related text) to phenotype the breast cancer cells. To this end, we have delineated a set of parameters that are categorized into two groups. The first group is related to the MS of the fluctuations over 100 s intervals and includes the cell-averaged MS, the number of high activity regions, and the mean area of these regions (Figure 5). The second group pertains to the cell-averaged best-fitting parameters of the linear fit of PSDs in log-log scale, specifically the power amplitude at 0.01 Hz and the slope (Figure 6). We start our discussion with the first group of parameters. Firstly, we found that the root mean square (RMS) of the intracellular fluctuations scales with cell malignancy (Figure 5A, top panel). Nontumorigenic MCF-10 cells exhibited a median RMS of 30 fg/μm^2^. In contrast, low-invasive MCF-7 cells showed a 30%–40% increase in the fluctuations and highly invasive MDA-MB-231 cells displayed fluctuations that were 3.7 times higher. The effect of ATP depletion on the intracellular fluctuations is intriguing. Contrary to expectations, the intracellular fluctuations increased by 50% in normal MCF-10A cells. This change was minor in low-invasive cancerous MCF-7 cells, whereas the fluctuations in MDA-MB-231 cells, in turn, decreased by approximately 55%. To understand these seemingly contradictory effects, we consider Hooke’s law, which governs the displacement fluctuations of intracellular components within the studied frequency range. Thus the RMSs of the displacement (x) and the stochastic forces (F) are related by ${\left\langle x^{2}\right\rangle }^{\frac{1}{2}}\approx \frac{{\left\langle F^{2}\right\rangle }^{\frac{1}{2}}}{\left|k\right|}$, where k is the complex local stiffness. The intracellular stiffness is primarily determined by the cytoskeleton, which relies on ATP hydrolysis for the assembly/disassembly of its filament fibers and for tensioning and crosslinking of the filament fiber mesh by specific motors.^61^^,^^62^^,^^63^ Recent research has established a close connection between cell stiffness and metabolism.^14^ This mechanobiological connection is altered in cancer, although in different ways depending on the cell invasiveness. In particular, it was found that ATP depletion made benign MCF-10-A cells four times softer and metastatic MDA-MB-231 cells 2.3 times softer. In contrast, low-invasive cancerous MCF-7 cells experienced minimal perturbation in stiffness. The effect of ATP depletion on the magnitude of intracellular fluctuations can thus be seen as a balance between the increase of passive fluctuations when the cells soften and the decrease of active fluctuations due to the lack of “fuel.” In MCF-10A cells, the former factor dominates, while the latter prevails in MDA-MB-231 cells. This result is consistent with the higher energy demands of metastatic cancerous cells compared to “normal” cells. Low-invasive cancerous MCF-7 cells represent an anomalous case where both the fluctuations and stiffness are minimally affected by ATP depletion.^14^ This suggests that these cells whose fate is proliferation in the primary tumor may have mechanisms to maintain their function in energy-deprived environments, highlighting the cancer metabolic plasticity at their different stages.^33^^,^^34^^,^^64^

We now turn our attention to the spatial heterogeneity of the intracellular fluctuations. We found significant differences in the number of highly active regions (hotspots) and their size across different cell lines (Figure 5A, middle and bottom panels). In MCF-10A cells, we observed a median of 300 highly active regions with a mean area of 0.4 μm^2^. Interestingly, in cancerous MCF-7 cells, the number of highly active regions decreased by approximately 20%, while the mean area increased by 40%. The most pronounced change was observed in metastatic cells, where the number of highly active regions reduced 6-fold and the area increased almost 5-fold. Under conditions of ATP depletion, we observed a significant decrease in the number of highly active regions in MCF-10A and MCF-7 cells, by 65% and 45%, respectively. Additionally, the area of these regions was reduced by approximately 50%. In contrast, for metastatic MDA-MB-231 cells, where activity was highly localized in specific regions of the cell, ATP depletion led to the disaggregation of these regions. This resulted in a 2-fold increase in the number of highly active regions and a 5-fold decrease in their size. Our findings lend further support to our conclusion that cancer cells meet their high energy demands by localizing ATP-dependent processes within specific cellular regions, rather than enhancing activity uniformly throughout the cell. This trend is particularly evident in metastatic cancer cells, indicating a shift in the spatial organization of intracellular activity as cells progress from a benign to a malignant state. This is further substantiated by ATP depletion experiments, which reveal a more uniform distribution of dominant passive fluctuations within the cells.

We summarize the phenotypic potential of the parameters associated with the magnitude of the intracellular fluctuations and their spatial distributions in Figures 5B and 5C. A higher degree of malignancy in cancer cells is characterized by an increased amplitude of the intracellular mass fluctuations and a larger degree of nucleation of intracellular activity, evidenced by fewer, yet larger, highly active regions. The data cloud and the corresponding probability density function of these parameters clearly differentiate the highly invasive MDA-MB-231 cells from the less-invasive cancerous MCF-7 cells and the benign MCF-10A cells. However, some overlap is observed between the benign MCF-10A and MCF-7 cells, which is likely due to the lower metabolic demands of MCF-7 cells due to their low invasiveness. Preliminary classification of our data using machine learning techniques yields prediction specificities of 72%, 76%, and 99% for MCF-10A, MCF-7, and MDA-MB-231 cells, respectively (see STAR Methods). The high specificities underscore the robust biological foundation of the chosen parameters for cell phenotyping.

We finally analyze the cell-averaged best-fitting parameters of the linear fit of the pixel PSDs in log-log scale, specifically the power amplitude at 0.01 Hz and the slope (Figure 6). The power amplitude of the intracellular fluctuations followed a trend similar to the MS of the fluctuations, scaling with cell malignancy (Figure 6A). However, a discrepancy arises when considering the effect of ATP depletion in MCF-10A cells. ATP depletion resulted in a 30% decrease of the power amplitude, while the MS increased by 50%. This discrepancy can be attributed to the fact that the MS integrates the dry mass fluctuations from 0.01 to 10 Hz, whereas the power amplitude only captures the fluctuation intensity at the lowest frequency, 0.01 Hz. Our attention is particularly drawn to the logarithmic slope, which provides valuable insights into the nature of intracellular fluctuations (Figure 6B). The mean logarithmic slopes are found to scale with cell malignancy, measuring 1.3 ± 0.1 for nontumorigenic MCF-10A cells, 1.4 ± 0.1 for low-invasive cancerous MCF-7 cells, and 1.6 ± 0.1 for metastatic MDA-MB-231 cells. Interestingly, these slopes decreased when the cells are deprived of energy and nutrients, dropping to 1.0 ± 0.1 in MCF-10A cells, 1.22 ± 0.15 in MCF-7 cells, and 1.30 ± 0.15 in MDA-MB231 cells. Despite the slopes being near 1 in all the cell lines, they still increase with cell malignancy.

These findings indicate that the logarithmic slope of the PSDs serves as reliable indicator of cell metabolism, correlating with cell malignancy. It is important to note that these results deviate from previous studies based on particle-tracer microrheology, which showed cell activity as a two-state system that was switched “on” in normal conditions, with tracer PSDs logarithmic slope near 2, and “off” in energy-deprived conditions with logarithmic slopes near 1.^35^^,^^36^^,^^57^^,^^60^ In this study, we demonstrate that active intracellular fluctuations are not ubiquitous within the cell but are localized and produced in accordance with the cell energy demands. Normal cells are closer to a state of thermal equilibrium than cancerous cells. Metabolic reprogramming in cancer alters the metabolic pathways to meet the specific energy needs of cancer cells at various stages of proliferation, invasion, and metastasis, progressively shifting the cell to a state increasingly distant from equilibrium. Under energy-deprived conditions, all cell lines tend to gravitate toward a state of equilibrium (indicated by a slope near 1). However, cancerous cells exhibit a slight deviation from this equilibrium, a deviation that intensifies with increasing malignancy. This behavior is attributed to the metabolic reprogramming that endows cancerous cells the ability to acquire necessary nutrients from nutrient-poor environments.^33^^,^^34^

The signatures of cell malignancy observed in the mean fitting parameters of the PSDs are summarized in Figure 6B. An increase in malignancy is associated with an increase in the power of the intracellular fluctuations and a higher logarithmic slope. The data cloud and the corresponding probability density function of these parameters distinctly differentiate the highly invasive MDA-MB-231 cells from the benign MCF-10A cells. The MCF-7 data falls between the MCF-10A and MDA-MB-231 data, with some overlap with both groups. We also carried out an initial machine learning-based classification of the data, which resulted in prediction specificities of 80%, 77%, and 86% for MCF-10A, MCF-7, and MDA-MB-231 cells, respectively. Notably, machine learning phenotyping based on the selected five parameters used here for describing the intracellular mass fluctuations, RMS of the fluctuation over 100-s intervals, number and size of the highly active regions, and the PSD fitting parameters, enhanced the specificity of the machine learning-based predictions to 99.9%, 99.0%, and 99.1% for MCF-10A, MCF-7, and MDA-MB-231 cells, respectively.

## Discussion

In this study, we have developed a computational method that significantly minimizes temporal phase noise in QPI. This enhancement allows for the precise quantification of stochastic intracellular dry mass fluctuations. By applying this advanced method alongside a newly developed image analysis technique to breast cancer cell lines, we have discovered metrics within these minor fluctuations that are strong indicators of both malignancy and metabolic condition. Our approach exceeds existing methodologies in gauging the phenotypic effects of cancer genetic variations and treatment responses. Fluorescence microscopy has traditionally been the preferred method for cell phenotyping due to its targeted visualization of cancer biomarkers.^7^^,^^8^ Nevertheless, it is constrained by the limited availability of specific and sensitive biomarkers, the necessity for fluorophore binding, and susceptibility to phototoxicity and photobleaching, which restricts the duration of observations. To overcome these drawbacks, label-free vibrational spectroscopy and microscopy, such as infrared and Raman spectroscopy, have been used.^65^^,^^66^^,^^67^ These methods offer chemical specificity in non-aqueous environments; however, their effectiveness is significantly reduced in aqueous conditions due to water’s obscuring effect, often necessitating the use of potentially harmful high-powered lasers for *in vivo* studies or *ex vivo* measurements on fixed cells. Additionally, these techniques are unable to monitor cellular dynamics and are characterized by low throughput. QPI, available in various modalities, enables precise morphological evaluations of cancer cells and the unique ability to monitor the dry mass of cells in real-time.^15^^,^^16^^,^^17^^,^^18^^,^^19^^,^^20^^,^^21^^,^^23^^,^^24^^,^^25^^,^^26^^,^^27^^,^^28^^,^^29^^,^^30^^,^^31^^,^^32^ While deep learning-assisted morphological analysis is highly specific for cell phenotyping, it struggles to link morphology with fundamental cancer hallmarks. Measuring the dry mass of cells over extended periods facilitates the investigation of cell growth and proliferation, which are primary targets of cancer genetic variants and therapeutic drugs. This method allows for the label-free, noninvasive, and high-throughput study of a broad array of genetic conditions and therapeutic protocols.^31^^,^^32^ However, one of the main limitations of these assays is the lengthy analysis time required, ranging from 20 h to several days. Our methodology offers rapid and accurate phenotyping based on cancer deregulated metabolism in just a few minutes. Previously, such measurements were exclusive to microrheology techniques, where fluorescence particles injected into the cytoplasm were tracked at 10 ms intervals for a few minutes.^35^^,^^36^^,^^37^ Although highly sensitive, these techniques provide only localized information at the tracer particles’ location. Moreover, these tracers can disrupt normal cellular functions, and fluorescence tracking is subject to the limitations of phototoxicity and photobleaching. Our technique is noninvasive, high-throughput, and distinctly capable of imaging metabolic activity throughout the cell. The technique introduced herein offers insights that refute the traditional paradigm asserting that living cells function in a state markedly distant from equilibrium. We have identified regions where mass fluctuations mirror the inherent thermal forces present in inert media, as well as areas of intense activity driven by ATP hydrolysis. These observations suggest that as cells navigate the complex processes of tumorigenesis, proliferation, and metastasis, they strategically enlarge these active zones to accommodate their increasing energy requirements. The specificity of our method can be enhanced by combination with aforementioned QPI methods for cell phenotyping. We foresee that integration of these advancements in QPI with single-cell genomics and genetic manipulation can unveil the phenotypic effects associated with cancer genetic variants and also serves as a critical tool for the evaluation of therapeutic strategies, addressing an acute need in cancer genetics and precision oncology.^1^^,^^2^^,^^3^^,^^20^

### Limitations of the study

Our study demonstrates that QPI combined with computational methods for spatial decorrelation of temporal phase noise provides a unique asset for studying stochastic intracellular fluctuations. The spatiotemporal patterns of these fluctuations encode sensitive indicators of the power and nature of these fluctuations, revealing the metabolic state of the cell. Our results indicate that metabolic activity correlates with cancer malignancy in breast cancer cells, offering a potential diagnostic tool for assessing cellular aggressiveness. While the technique and results are promising, the clinical impact requires validation in more complex and diverse samples. Future investigations will expand on this work by incorporating a broader range of cell types and conditions to ensure the generalizability of our findings. Longitudinal studies are also planned to monitor the evolution of intracellular fluctuations over time, providing insights into disease progression and treatment efficacy. The planned use of manipulative genetics, such as CRISPR-Cas9 and RNA interference, will allow us to fine-tune cellular aggressiveness and assess its impact on intracellular fluctuations, further strengthening the scientific rigor of our study.

## Resource availability

### Lead contact

Further information and requests for resources should be directed to and will be fulfilled by the lead contact, Javier Tamayo (javier.tamayo@csic.es).

### Materials availability

This study did not generate any new unique reagents.

### Data and code availability

• The datasets in this study are available on request.
• The code snippets corresponding to the noise decorrelation algorithm supporting the current study is available in Methods S1.
• Any additional information required to reanalyze the data reported in this work paper is available from the lead contact upon request.

## Acknowledgments

This work was partially supported by the Spanish Science and Innovation Ministry through projects RTI2018-099369-B-I00 (related doctoral fellowship [FPI] PRE2019-087448), AES (DTS21/00136)/ISCIII/EU/FEDER, “CELLBIOPHYS” (PID2021-126993OB-I00/MCIN/AEI/10.13039/501100011033/FEDER, UE), “ONCOLIGHT” (PDC2022-133503- I00/MCIN/AEI/10.13039/501100011033/European Union/Next Generation EU/PRTR), and “ONCODEEPLASM” (PLEC2021- 007892/MCIN/AEI/10.13039/501100011033/European Union/Next Generation EU/PRTR) and by 10.13039/100012818Comunidad Autónoma de Madrid (Y2020/BIO- 65194 scCANCER-CM). This work has also been supported by private FERO Foundation and Elena Torres Cancer Association funding. We acknowledge the service from the Micro and Nanofabrication Laboratory and X-SEM laboratory at IMN-CNM funded by the 10.13039/100012818Comunidad Autónoma de Madrid (project S2018/NMT-4291 TEC2SPACE) and by 10.13039/501100003329MINECO (project CSIC13-4E-1794 with support from FEDER, FSE).

## Author contributions

A.C. performed all the imaging experiments; M.L.Y. and P.M.K. performed the biochemistry and cell culture and contributed to the biological interpretation of the results; A.C., C.M., V.P.-B., J.T., and J.J.R. developed the noise suppression method and image analysis methodology; M.C., M.M., and J.T. conceived and designed the experiments; J.T. wrote the manuscript; all authors discussed results and edited the manuscript.

## Declaration of interests

A.C., M.L.Y., C.M., V.P.-B., P.M.K., J.J.R., M.C., and J.T. wish to declare that they have applied for a patent related to the work presented in this paper. The patent application number 202330868, pertaining to “METHOD AND SYSTEM FOR IMAGE ANALYSIS FOR DETERMINATION OF INTRACELLULAR FLUCTUATIONS,” in the name of the Spanish National Research Council (CSIC), was filed with the Spanish Patent and Trademark Office on October 20, 2023. This patent application has not altered our adherence to Cell journal’s policies on data and material sharing.

## STAR★Methods

### Key resources table

**Table:** 

REAGENT or RESOURCE	SOURCE	IDENTIFIER
**Chemicals, peptides, and recombinant proteins**		

Dulbecco’s Modified Eagle’s Medium – high glucose	Thermo-Fisher	Cat#: 41965039
Fetal Bovine Serum	Sigma-Aldrich	Cat#: F7524
Penicillin-Streptomycin	Sigma-Aldrich	Cat#: P4333
0.05% Trypsin-EDTA	Sigma-Aldrich	Cat#: T3924
DMEM/F12	Thermo-Fisher	Cat#: 11320033
Horse serum	Sigma-Aldrich	Cat#: H1270
Epidermal growth factor	Thermo-Fisher	Cat#: PHG0311
Hydrocortisone	Sigma-Aldrich	Cat#: H0888
Cholera toxin	Sigma-Aldrich	Cat#: C8052
Insulin	Sigma-Aldrich	Cat#: I1882

**Experimental models: Cell lines**		

MDA-MB-231	ATCC	Cat#: HTB-26
MCF-7	ATCC	Cat#: HTB-22
MCF-10A	ATCC	Cat#: CRL-10317

**Software and algorithms**		

Koala	Lyncée-Tec	https://www.lynceetec.com/ N/A
Origin	Origin-Lab	https://www.originlab.com RRID:SCR_014212
Wolfram Mathematica	Wolfram-Research	https://www.wolfram.com/mathematica/ RRID:SCR_014448
Python	Python Software Foundation	https://www.python.org/ RRID:SCR_008394

### Experimental model and study participant details

#### Cell culture

The cell lines MCF-7 (female), MDA-MB-231 (female), and MCF-10A (female) were obtained from the American Type Culture Collection (ATCC®, USA). MCF-7 and MDA-MB-231 cells were cultured in Dulbecco’s modified Eagle’s medium (DMEM, Gibco, Life Technologies Corporation, Rockville, MD, USA) supplemented with 10% fetal bovine serum (FBS), 500 U/mL penicillin, and 0.1 mg/mL streptomycin. Meanwhile, MCF-10A cells were cultured in DMEM/F12 medium (Gibco) supplemented with 5% horse serum, 20 ng/mL epidermal growth factor, 0.5 μg/mL hydrocortisone, 100 ng/mL cholera toxin, 10 μg/mL insulin, and 500 U/mL penicillin and 0.1 mg/mL streptomycin. Cells were maintained in a humidified incubator at 37°C with 5% CO2 (Eppendorf, Germany). Cell lines subjected to high passage numbers often undergo alterations in morphology, growth rates, protein expression, and cell signaling. To guarantee the reproducibility of our experiments, all cell cultures used in this work were limited to a maximum of 10 passages.

### Method details

#### Digital holographic microscopy

Experiments were performed using a Transmission DHM® T-2100 from Lyncee Tec (Switzerland), equipped with a 40x/0.75 NA objective (Leica N-Plan). To mitigate the impact of external mechanical vibrations, the digital holographic microscope was mounted on an active optical table (Newport, Guardian® Active Isolation Workstation) to guarantee a stable environment for precise and accurate measurements. In each measurement, holograms were acquired at intervals of 0.05 seconds over a total time of 100 seconds. Phase images were reconstructed from the acquired holograms using Koala® software (Lyncee Tec, Switzerland). To enhance image quality, a reference hologram was obtained before the measurements. Subsequently, this reference hologram was subtracted from each hologram during the reconstruction process. For a precise and comprehensive analysis of the acquired data, image processing and statistical analysis were carried out through custom algorithms developed in Wolfram Mathematica® (Wolfram Research).

#### Sample preparation

Since mechanical properties are significantly influenced by intracellular contacts, DHM measurements were conducted when the cells reached the desired confluence or a steady state. This was achieved by seeding the cells at a density of $2\times 10^{5}$ cells/mL on 35 mm cell culture plates (Corning® CellBIND® Surface) for 24–36 hours. Following that, cells were detached from the dish using a trypsin-EDTA mixture and subsequently sub-cultured. This process involves adding 120 μL of the resuspended culture into optical glass perfusion chambers with a channel height of 0.8 mm (μ-Slide l Luer Glass Bottom, ibiTreat, Ibidi). The cells are then incubated in the chambers for 24 hours before measurement. To ensure cell viability during the experiments, they were performed within a humidified incubator maintained at 37°C and with 5% CO_2_ (Live Cell Instrument, CU-501).

*ATP depletion* - Complete depletion of ATP requires the inhibition of both oxidative and glycolytic metabolisms. This was achieved by culturing cells in a glucose-free medium supplemented with 20 mM NaN_3_ and 5 mM 2-deoxy-D-glucose. Following a 1-hour incubation, DHM measurements were conducted. It is noteworthy that the administered doses and incubation times were intentionally set to significantly exceed the threshold needed to reach a saturation response from the cells while ensuring the maintenance of their viability throughout the experimental process.

#### Image analysis

The image datasets are processed and analyzed following these steps:

1. OPD images were acquired at 20 frames per second during 100-s intervals. The cell temporal series were isolated from the background by designing masks for each cell and for the background at discrete regions well-separated from the cells. The spatially-averaged OPD at these background regions are used as references for retrieval of the cell-intrinsic OPD fluctuations. The cell masks were kept constant to capture the local mass fluctuations, while the center position of the mask was recalculated every second for correcting any potential thermal drift.
2. The decorrelation method, as described in the main text, was applied to the temporal series of the OPD images in order to isolate the cell intrinsic fluctuations from the spatially-correlated noise. After that, the OPDs at the cell and the references were treated as uncorrelated. The power (or square) of the OPD signal at each cell pixel can then be represented as the sum of the power of the OPD due to intracellular fluctuations and the power of the spatially uncorrelated noise. We estimated the power of the intracellular fluctuations for each pixel within the cell by subtracting the OPD noise power, which was derived from the references. The analysis only considers those pixels of the cell with activity well-above the background noise.
3. After application of the denoising methods, the mean-square images of the cells were calculated. The cell pixels were arranged in descending order of MS values. Pixels were then selected until the cumulative MS reaches a threshold value, which in this case is set to 68.2% of the total MS of the cell. The resulting image reveals clusters of connected pixels where the fluctuations are more localized. For each cell, we calculate, the average of the MS images, the number of clusters, and mean area of the clusters.
4. The Fast Fourier Transform (FFT) is calculated for each pixel in both the cells and the background regions. The Power Spectral Density (PSD) is then derived from the square of these FFT values. The PSD floor is determined by the average PSD in the background regions. The PSDs of the cell pixels are then filtered, retaining only for subsequent fitting analysis those where the amplitude exceeds 25% of the PSD floor for at least 70% of the frequencies (Figure S3). The differential PSDs are fitted to the following function, $f\left(\omega \right)=\frac{A}{\omega^{m}}+B$, where A is the amplitude parameter, m is the logarithmic slope and the constant B is a constant correction parameter that mimics the transition from active to thermal fluctuations at high frequencies. The logarithmic slope is used in this study to estimate the nature of the intracellular stochastic activity (see Methods S1 and Figure S4). For each cell, we calculated the average of the best fitting parameters.

### Quantification and statistical analysis

A series of independent experiments were conducted: 14 for MCF-10A cells under normal conditions, 12 for MCF-10A cells under ATP-depleted conditions, 11 for MCF-7 cells under both normal and ATP-depleted conditions, 10 for MDA-MB-231 cells under normal conditions, and 12 for MDA-MB-231 cells under ATP-depleted conditions. From these experiments, a total of 217 MCF-10A cells, 267 MCF-10A cells under ATP depletion, 150 MCF-7 cells, 250 MCF-7 cells under ATP depletion, 132 MDA-MB-231 cells, and 113 MDA-MB-231 cells under ATP depletion were analyzed. In the spatial cluster and PSD analyses, 10-20% of these data were excluded. Specifically, data falling below Q1-1.5×IQR or above Q3+1.5I×QR were excluded, where Q1 and Q3 represents the first and third quartiles, respectively and IQR=Q3-Q1, the interquartile range. These outliers are usually found in the images with the presence floating bubbles or debris near the region of interest.

A machine learning approach was employed for the initial classification of the data in our study. The data was partitioned into two halves: one half was utilized for training the model, while the other half was reserved for evaluation. Upon examination of various models, it was found that the logistic regression model was optimal for our classification task. The logistic regression model was trained on the training data, and the resulting classifier was applied to the evaluation data. The performance of the classifier was assessed based on its specificity, which measures the proportion of correct predictions of a particular class among all instances that are actually of that class.
