# Supplementary material for: Rapid mechanical phenotyping of breast cancer cells based on stochastic intracellular fluctuations
Source: iScience. 2024 Oct 4;27(11):110960. doi: 10.1016/j.isci.2024.110960 (PMC11530848; doi:10.1016/j.isci.2024.110960)
Supplement: Document S1. Methods S1 [file mmc1.pdf]

iScience, Volume *27*

Supplemental information

Rapid mechanical phenotyping of breast cancer cells based on stochastic intracellular fluctuations

Álvaro Cano, Marina L. Yubero, Carmen Millá, Verónica Puerto-Belda, Jose J. Ruz, Priscila M. Kosaka, Montserrat Calleja, Marcos Malumbres, and Javier Tamayo

Numerical and theoretical methods

Method for suppression of spatially-correlated phase noise

We define a time-stochastic function $s(x, y, t)$ with a time-average zero defined in a 2D domain Ω that can be described as,

$$s(x, y, t) = u(x, y, t) + f(x, y, t) \tag{S1}$$

where $u(x, y, t)$ is a function that is zero everywhere except in a well-known subdomain $\Omega_C \subset \Omega$, and $f(x, y, t)$ is a function with a smooth spatial variation over the domain Ω. We assume that u and f are uncorrelated in time. The function $f(x, y, t)$ can generally be expanded in power series of x and y as,

$$f(x, y, t) = \sum_{m,n} C_{mn}(t) x^m y^n \tag{S2}$$

where $C_{mn}(t)$ are time-stochastic coefficients. The goal is to retrieve the unknown stochastic signal $u(x, y, t)$ from the detected stochastic signal $s(x, y, t)$. To this end, a set of subdomains $\Omega_i \subset \Omega$ that do not intersect with the domain Ω_C ($\Omega_C \cap \Omega_i = \emptyset \; \forall i$) are selected. The spatial average of the detected stochastic signal over each of these subdomains is given by,

$$r_i(t) = \sum_{m,n} C_{mn}(t) \langle x^m y^n \rangle_{\Omega_i} \tag{S3}$$

From this expression, it can be derived that if the set of subdomains is spatially well distributed over the entire domain Ω, the temporal fluctuation of the function $f(x, y, t)$ can be approximated by,

$$f(x, y, t) \approx \sum_{i=1}^{N} \alpha_i(x, y) r_i(t) \tag{S4}$$

where $\alpha_i(x, y)$ are general functions to be determined and *N* is the number of subdomains. In order to obtain the functions $\alpha_i(x, y)$, we calculate the time correlations between the detected fluctuation signal $s(x, y, t)$ and its spatial average over each of the subdomains Ω_i,

$$\rho_j(x, y) = \int u(x, y, t) r_j(t)\, dt + \sum_{i=1}^{N} \alpha_i(x, y) \int r_j(t)\, r_i(t) dt \tag{S5}$$

Since the functions u and r_i are uncorrelated in time with zero mean, the first integral vanishes and we obtain the following linear system of equations for the functions $\alpha_i(x, y)$,

$$\rho_j(x, y) = \sum_{i=1}^{N} \alpha_i(x, y) \int r_j(t)\, r_i(t) dt \tag{S6}$$

Once we obtain the functions $\alpha_i(x, y)$ from this system of equations, we can estimate the unknown stochastic signal $u(x, y, t)$ as,

$$u(x, y, t) \cong s(x, y, t) - \sum_{i=1}^{N} \alpha_i(x, y) r_i(t) \tag{S7}$$

It should be noted that the selection of the subdomains Ω_i is crucial for obtaining an accurate estimation of the signal $u(x,y,t)$. These subdomains must be well-distributed in the domain Ω to capture the spatial dependence of the spatially-correlated noise, $f(x,y,t)$. However, since the function $f(x,y,t)$ is unknown, there might be some situations where the system of equations (S6) has determinant very close to zero due to redundant information from the subdomains. This can lead to significant errors in the calculation of the coefficients $\alpha_i(x,y)$. One way to solve Eq. (S6) is to use advanced numerical methods like pseudo-inverse calculation. Another way is to solve the system iteratively, neglecting the correlations between $r_i(t)$ and $r_j(t)$ for $i \neq j$ in every step. In this way, we start by approximating the functions, $\alpha_j(x,y) \approx \frac{\rho_j(x,y)}{\int r_j(t)^2 dt}$, which leads to an initial estimation of the unknown stochastic signal $u(x,y,t)$ by using equation (S7). We then replace the function $s(x,y,t)$ by the estimation of $u(x,y,t)$, and repeat the process iteratively up to achieving convergence.

In order to validate the decorrelation method, we have performed numerical simulations of a stochastic signal in the domain Ω_C hidden by a background of spatially-correlated random noise. We have chosen three subdomains for the background, Ω_1, Ω_2 and Ω_3 (**Fig. S1a**). We assume that the spatially correlated noise is produced by a time-stochastic tilt of the surface induced by mechanical vibrations given by,

$$f(x,y,t) = a(t)x + b(t)y + c(t) \tag{S8}$$

where $a(t)$, $b(t)$ and $c(t)$ are here described by Wiener processes (**Fig. S1b**). For the stochastic signal in the Ω_C domain, we have chosen a linear combination of orthonormal functions given by,

$$\psi_{mn}(x',y') = \sin\left(m\pi\left(x' - \frac{1}{2}\right)\right)\sin\left(n\pi\left(y' - \frac{1}{2}\right)\right) \tag{S9}$$

where x', y' are coordinates centered at Ω_C and normalized to the dimensions of Ω_C, and m and n are integers greater than or equal to 1. The stochastic signal is here modeled as,

$$u = d_{21}(t)\psi_{21}(x',y') + d_{13}(t)\psi_{13}(x',y') + d_{31}(t)\psi_{31}(x',y') + d_{33}(t)\psi_{33}(x',y') \tag{S10}$$

where $d_{21}(t)$, $d_{13}(t)$, $d_{31}(t)$ and $d_{33}(t)$ are described by Wiener processes. **Figure S1c** shows the resulting stochastic signal $s(x,y,t)$ averaged over the background domains, $r_1(t)$, $r_2(t)$ and $r_3(t)$, and averaged over the domain Ω_C, referred to as $p(t)$. The signal $p(t)$ becomes indiscernible from the background signals due to the spatially correlated noise, a situation that mirrors our experimental observations. Following the application of the decorrelation method, the retrieved signal $p(t)$ fits remarkably well with the unperturbed signal within the domain Ω_C. The deviation between the retrieved and original signals is approximately 10%. The discrepancy is attributed to the existing correlation between the signal of interest $u(x,y,t)$ and the spatially correlated noise $f(x,y,t)$, which is not accounted for in the decorrelation method. This oversight occurs because both the signal of interest

and the spatially correlated noise are characterized by stochastic Wiener processes, which cannot be entirely decorrelated within a finite time span.

Code snippets of the decorrelation algorithm program

Here, we provide the key code snippets in Python used in our decorrelation algorithm. In order to run the code, a Python interpreter along with the Numpy library must be correctly installed. Let us assume that $s(x, y, t)$ is the original image contained in the domain Ω. Since we have a limited number of pixels and a limited number of time frames, we should express $s(x, y, t)$ in a discrete form as $s(i, j, k)$, where i, j are integers that represent the row and column of each pixel, and k represents the time frame number. This set of images must be loaded into a Numpy array S of dimensions nx by ny by nt, where nx and ny are the number of pixels of the image along x and y directions respectively and nt is the number of time frames. We select the set of N background subdomains Ω_n where no cells are present, as shown in **Fig. S1a**. These background subdomains must be transformed into binary arrays with the same size of each frame image. The decorrelation algorithm proceeds with the following steps:

1. Calculate the fluctuation of the signal $\Delta s(i, j, k)$:
   Python code:
   ```
   dS = S-np.mean(S,axis=2)[:,:,np.newaxis]
   ```
2. Calculate the spatial average of the fluctuation over each of the background subdomains for each time frame: $r_n(k) = \frac{1}{M_n}\sum_{i,j} \Delta s(i, j, k)$ where this sum is performed over the pixels of the subdomain Ω_n and M_n is the total number of pixels contained in such subdomain.
   Python code:
   ```
   r_array = np.zeros((N,np.shape(dS)[2]))
   for i in range(N):

          r_array[i,:] =
          np.sum(dS*subs_mask[i][:,:,np.newaxis],axis=(0,1))/np.sum(subs_mask
          [i])
   ```

   Here `subs_mask` is a list that contains the binary arrays for the substrates.

3. We calculate the correlation matrices $\rho_n(i, j)$ corresponding to each of the background subdomains as follows: $\rho_n(i, j) = \sum_{k=1}^{nt} r_n(k)\Delta s(i, j, k)$ where nt is the total number of time frames. These matrices are calculated over the entire image, i.e. the entire domain Ω.
   Python code:
   ```
   rho_list = []
   for i in range(N):
          rho_list.append(np.dot(dS,r_array[i,:]))
   ```

4. For each pixel i,j of the whole image, we form the following system of N equations: $\rho_n(i,j) = \sum_{m=1}^{N} \alpha_m(i,j) \sum_{k=1}^{nt} r_n(k) r_m(k)$.
5. We solve the system of equations for each pixel to obtain each of the matrices $\alpha_m(i,j)$. This system can be solved directly or by means of an iterative method as described before.
   Python code:

```
cross_r = np.dot(r_array,r_array.T)
b = np.transpose(np.asarray(rho_list),(1,2,0))
matrix_inv = np.linalg.pinv(cross_r)
alpha = np.matmul(b, matrix_inv.T)
```

6. Once we have obtained the matrices $\alpha_m(i,j)$ we calculate the final decorrelated fluctuation as: $\Delta u(i,j,k) = \Delta s(i,j,k) - \sum_{m=1}^{M} \alpha_m(i,j) r_m(k)$
   Python code:

```
dSnoise = np.zeros(np.shape(dS))
for i in range(N):
       dSnoise += alpha[:,:,i][:,:,np.newaxis]*r_array[i,:]
dS = dS-dSnoise
```

Quantification of temporal phase noise

In quantitative phase imaging (QPI), the temporal phase sets the limit of QPI for quantifying dry mass variations of living cells. This noise is typically determined by calculating the standard deviation of the phase in image regions devoid of cells[1–4]. In our study, we selected several background regions that were well-separated from the cells and similar in size to the cells. To reduce the temporal phase noise, spatial and time averaging of the phase has been proposed. As our work focuses on rapid intracellular fluctuations, time averaging is not suitable for fluctuations on a millisecond scale. Consequently, the phase noise is governed by the spatial scale of the fluctuations. Furthermore, the relationship between the phase noise and the spatial bandwidth reveals the nature of the noise. To accurately quantify the phase noise and analyze the effect of the decorrelation algorithm, we calculated the standard deviation of the optical path distance ($OPD = \frac{\lambda}{2\pi}\phi(x,y)$, where ϕ is the phase) as a function of the averaging area in the background regions and the cells as shown in Fig. 2 in the main text (**Figure S2**). Prior application of the decorrelation algorithm, the standard deviation of the OPD per pixel (0.28 µm pixel size) in the background regions is 2.7 nm. Spatial averaging reduces this noise until the averaging area radius reaches about 1-1.2 µm (approximately 36 pixels). Beyond this point, the OPD noise asymptotically approaches 1.1 nm for larger spatial averaging. The phase noise exceeds the standard deviation of the cell fluctuations for an averaging area radius above 2 µm, as a consequence of the spatially

correlated phase noise. This results in a minimum detectable variation of the dry mass of the cells (with spatial averaging of approximately 700 µm², equivalent to 9000 pixels) of about 4 pg[1–4]. After the application of the decorrelation algorithm, the phase noise in the background regions becomes white, benefiting from spatial averaging with the number of pixels approximating $1/\sqrt{N}$. While the per-pixel OPD noise reduces by 20%, for features with a 1 µm radius, the OPD noise reduces threefold from 1.2 nm to 0.4 nm. For cell integration areas, the OPD noise reduces further to 0.03 nm, setting the minimum detectable cell mass variation at about 0.1 pg, a 40-fold improvement. Notably, the cell fluctuations are consistently above the noise for all the averaging radii.

Model for fitting stochastic intracellular fluctuations

We begin by the examination of the stochastic fluctuations of a protein within the cell. The protein position is governed by the differential equation of a stochastic damped harmonic oscillator[5–10],

$$m\ddot{x}(t) + \gamma\dot{x}(t) + kx(t) = \xi(t) + f_a(t) \tag{S11}$$

where m is the mass of the protein, k is the local spring constant of the cell at the position of the protein, γ is the local damping coefficient, ξ represents the random thermal forces acting on the protein, and f_a is the stochastic active force due to the uncorrelated action of multiple motors. Under the assumption of cellular medium isotropy, it is posited that the fluctuations of protein are directionally independent. Cell viscoelasticity is described through a complex spring constant that follows power-law dependency with frequency given by,

$$k = k_0\left(i\frac{\omega}{\omega_0}\right)^{\beta} \tag{S12}$$

, where k_0 is the spring constant modulus at the arbitrary reference frequency ω_0 defined here as 2π Hz and β is the local power-law exponent that can vary between 0 (elastic solid) and 1 (viscous liquid). Multitude of studies underscore the universal nature of the power-law behavior in living cells[9–11]. This behavior spans a broad frequency spectrum, specifically between 0.01 and 1 KHz, within which, β generally varies between 0.05 and 0.3. Within the frequency range of our analysis, 0.01-10 Hz, the inertial and damping forces in Eq. (S11) are negligible relative to the viscoelastic force. By applying the Fourier transform to Eq. (S11), we compute the power spectral displacement (PSD) associated with the displacement fluctuations of the protein,

$$\langle|\hat{x}(\omega)|^2\rangle = \frac{\langle|\hat{\xi}(\omega)|^2\rangle + \langle|\hat{f}_a(\omega)|^2\rangle}{{k_0}^2\left(\frac{\omega}{\omega_0}\right)^{2\beta}} \tag{S13}$$

where ^ stands for the Fourier transform and ⟨ ⟩ for the ensemble average. Notice that Eq. (S13) assumes that the thermal and active forces are temporally uncorrelated. The PSD of the thermal force is obtained from the fluctuation-dissipation theorem[5–10],

$$\langle|\hat{\xi}(\omega)|^2\rangle = 4\frac{k_0\left(\frac{\omega}{\omega_0}\right)^{\beta}\sin\left(\frac{\pi}{2}\beta\right)}{\omega}k_BT \quad \text{(S14)}$$

where k_B is Boltzmann's constant, and T is the absolute temperature. The PSD of the stochastic active force is described by,

$$\langle|\hat{f}_a(\omega)|^2\rangle \approx \frac{{f_m}^2}{2}\frac{\tau}{1+(\tau\omega)^2} \quad \text{(S15)}$$

Eq. (S15) assumes that the molecular motors randomly generate step like forces of force $\pm f_m$ during a pulse time that follows a Poisson distribution with mean τ, referred to as processivity time[8–10]. Substituting Eqns. (S14) and (S15) into Eq. (S13), we obtain,

$$\langle|\hat{x}(\omega)|^2\rangle = \left(\frac{4\sin\left(\frac{\pi}{2}\beta\right)k_BT}{k_0\,\omega\left(\frac{\omega}{\omega_0}\right)^{\beta}} + \frac{{f_m}^2\tau}{2\,{k_0}^2\,(1+(\tau\omega)^2)\left(\frac{\omega}{\omega_0}\right)^{2\beta}}\right) \quad \text{(S16)}$$

We now examine Eq. (S16) to interpret the logarithmic slope of the PSD of protein displacements. We start by analyzing the asymptotic regimes where the fluctuations are dominated by thermal and active forces. In the thermal regime ($f_m = 0$), the PSDs exhibits a linear dependence with the frequency in the log-log scale, with negative slope of $1+\beta$. Conversely, the regime where active fluctuations prevail, the amplitude initially displays a weak decay with the frequency as $\sim\omega^{-2\beta}$ when $\omega \ll 1/\tau$, whereas this decay becomes more pronounced as the frequency increases, reaching $\sim\omega^{-2(1+\beta)}$ for $\omega \gg 1/\tau$. Proteins, in general, are subjected to both types of stochastic forces, leading to intermediate behaviors and more complex frequency-dependence (**Fig. S4a**). In the asymptotic limit of high frequencies, thermal fluctuations dominate over the active fluctuations, and the amplitude decays as $\sim\omega^{-(1+\beta)}$. In the lower frequency regime, the active fluctuations become more significant. Depending on the relative contribution of the thermal forces to the total fluctuations, different frequency dependences can be observed. Interestingly, the theoretical PSDs can be fitted by a simple phenomenological function (**Fig. S4b**),

$$f(\omega) = \frac{A}{\omega^m} + B \quad \text{(S17)}$$

, where A is the amplitude parameter, m is the logarithmic slope and the constant B is a constant correction parameter that mimics the transition from active to thermal fluctuations at high frequencies. The logarithmic slope used in this study to estimate the nature of the intracellular stochastic activity, increases as the active forces increase. The mean R-squared (coefficient of determination) is only below 1 by about 10^{-5}, indicating the goodness of the fitting. Although, the phenomenological model does not allow to extract quantitative parameters about the viscoelasticity such as k_0 and β, and about the active forces such as f_m and τ, the logarithmic slope serves as a reliable indicator of the metabolic activity. It is important to note that experimental data about the stochastic fluctuations of intracellular particle tracers also have significant instrumental noise[9,10]. Fitting this type of experimental data to the theory (Eq. (S16)) can lead to overfitting problems, resulting in degeneracy in the

fitting parameters. To circumvent this issue, researchers often make assumptions that involve ignoring either thermal or active fluctuations, contingent on the experimental circumstances. For instance, when cells are in a standard culture medium, thermal fluctuations are usually neglected, while in ATP depletion conditions, active fluctuations are typically disregarded.

We now connect the fluctuations of a single particle with the fluctuations of our observable, the dry mass surface density. Assuming that during the time of observation, synthesis and degradation of proteins is negligible, the local density of proteins, $n_p(\vec{r})$, is governed by the following continuity equation,

$$\frac{\partial n_p}{\partial t} = -\nabla(n_p \overrightarrow{v_p}) \quad \text{(S18)}$$

where $\overrightarrow{v_p}$ is the flow velocity vector. By applying Fourier transform to Eq. (S18), we calculate the PSD of the stochastic fluctuations in the local density of proteins,

$$\langle|\hat{n}_p(\vec{r},\omega)|^2\rangle = \left(\left(\frac{\partial n_p(\vec{r})}{\partial x}\right)^2 + \left(\frac{\partial n_p(\vec{r})}{\partial y}\right)^2 + \left(\frac{\partial n_p(\vec{r})}{\partial z}\right)^2\right)\langle|\hat{x}_p(\vec{r},\omega)|^2\rangle \quad \text{(S19)}$$

Since the displacement fluctuations are independent of the protein mass, Eq. (S19) can directly be expressed in terms of the dry mass density ρ,

$$\langle|\hat{\rho}(\vec{r},\omega)|^2\rangle = \left(\left(\frac{\partial \rho(\vec{r})}{\partial x}\right)^2 + \left(\frac{\partial \rho(\vec{r})}{\partial y}\right)^2 + \left(\frac{\partial \rho(\vec{r})}{\partial z}\right)^2\right)\langle|\hat{x}_p(\vec{r},\omega)|^2\rangle \quad \text{(S20)}$$

By integrating Eq. (S20) in the z direction and plugin Eq. (S16) into Eq. (S20), we obtain the PSD of the dry mass surface density,

$$\langle|\hat{\sigma}(x,y,\omega)|^2\rangle = \left(\left(\frac{\partial \sigma(x,y)}{\partial x}\right)^2 + \left(\frac{\partial \sigma(x,y)}{\partial y}\right)^2\right)\int_0^{h(x,y)} \left(\frac{4\sin\left(\frac{\pi}{2}\beta(\vec{r})\right)k_B T}{k_0(\vec{r})\omega\left(\frac{\omega}{\omega_0}\right)^{1+\beta(\vec{r})}} + \frac{f_m(\vec{r})^2\tau(\vec{r})}{2\,k_0(\vec{r})^2\,(1+(\tau(\vec{r})\omega)^2)\left(\frac{\omega}{\omega_0}\right)^{2\beta(\vec{r})}}\right)dz \quad \text{(S21)}$$

where h is the height of the cell. The stochastic displacements of numerous proteins, on the order of approximately 10^5 per square micrometer, contribute to the fluctuations of the dry mass surface density. Each protein at a given position (x, y) can encounter varying degrees of viscoelasticity and active forces based on its z-position, thereby contributing to the dry mass density fluctuations in diverse magnitudes. As previously discussed, deciphering the

contributions of thermal and active fluctuations poses a significant challenge even for single intracellular particle tracers. In the context of surface dry mass density, this task becomes unfeasible. However, by fitting the experimental PSDs at each (x, y) position of the cell to the phenomenological model (Eq. (S17)), we can uncover the heterogeneity of the stochastic fluctuations throughout the cell via the fitting parameter - the logarithmic slope.

References

1. Zangle, T.A., and Teitell, M.A. (2014). Live-cell mass profiling: an emerging approach in quantitative biophysics. Nat Methods *11*, 1221–1228. https://doi.org/10.1038/nmeth.3175.

2. Nguyen, T.L., Pradeep, S., Judson-Torres, R.L., Reed, J., Teitell, M.A., and Zangle, T.A. (2022). Quantitative Phase Imaging: Recent Advances and Expanding Potential in Biomedicine. ACS Nano *16*, 11516–11544. https://doi.org/10.1021/acsnano.1c11507.

3. Aknoun, S., Savatier, J., Bon, P., Galland, F., Abdeladim, L., Wattellier, B.F., and Monneret, S. (2015). Living cell dry mass measurement using quantitative phase imaging with quadriwave lateral shearing interferometry: an accuracy and sensitivity discussion. JBO *20*, 126009. https://doi.org/10.1117/1.JBO.20.12.126009.

4. Liu, X., Oh, S., Peshkin, L., and Kirschner, M.W. (2020). Computationally enhanced quantitative phase microscopy reveals autonomous oscillations in mammalian cell growth. Proceedings of the National Academy of Sciences *117*, 27388–27399. https://doi.org/10.1073/pnas.2002152117.

5. Lau, A.W.C. (2003). Microrheology, Stress Fluctuations, and Active Behavior of Living Cells. Phys. Rev. Lett. *91*. https://doi.org/10.1103/PhysRevLett.91.198101.

6. Mizuno, D., Tardin, C., Schmidt, C.F., and MacKintosh, F.C. (2007). Nonequilibrium Mechanics of Active Cytoskeletal Networks. Science *315*, 370–373. https://doi.org/10.1126/science.1134404.

7. Wilhelm, C. (2008). Out-of-Equilibrium Microrheology inside Living Cells. Phys. Rev. Lett. *101*, 028101. https://doi.org/10.1103/PhysRevLett.101.028101.

8. MacKintosh, F.C. (2008). Nonequilibrium Mechanics and Dynamics of Motor-Activated Gels. Phys. Rev. Lett. *100*. https://doi.org/10.1103/PhysRevLett.100.018104.

9. Guo, M., Ehrlicher, A.J., Jensen, M.H., Renz, M., Moore, J.R., Goldman, R.D., Lippincott-Schwartz, J., Mackintosh, F.C., and Weitz, D.A. (2014). Probing the Stochastic, Motor-Driven Properties of the Cytoplasm Using Force Spectrum Microscopy. Cell *158*, 822–832. https://doi.org/10.1016/j.cell.2014.06.051.

10. Fodor, É., Guo, M., Gov, N.S., Visco, P., Weitz, D.A., and Wijland, F. van (2015). Activity-driven fluctuations in living cells. EPL *110*, 48005. https://doi.org/10.1209/0295-5075/110/48005.

11. Kollmannsberger, P., and Fabry, B. (2011). Linear and Nonlinear Rheology of Living Cells. Annual Review of Materials Research *41*, 75–97. https://doi.org/10.1146/annurev-matsci-062910-100351.
