# Supplementary material for: Rapid mechanical phenotyping of breast cancer cells based on stochastic intracellular fluctuations
Source: iScience. 2024 Oct 4;27(11):110960. doi: 10.1016/j.isci.2024.110960 (PMC11530848; doi:10.1016/j.isci.2024.110960)
Supplement: Document S2. Figures S1–S4 [file mmc2.pdf]

Supplemental information

Rapid mechanical phenotyping of breast cancer cells based on stochastic intracellular fluctuations

Álvaro Cano, Marina. L. Yubero, Carmen Millá, Verónica Puerto-Belda, Jose J. Ruz, Priscila M. Kosaka, Montserrat Calleja, Marcos Malumbres and Javier Tamayo

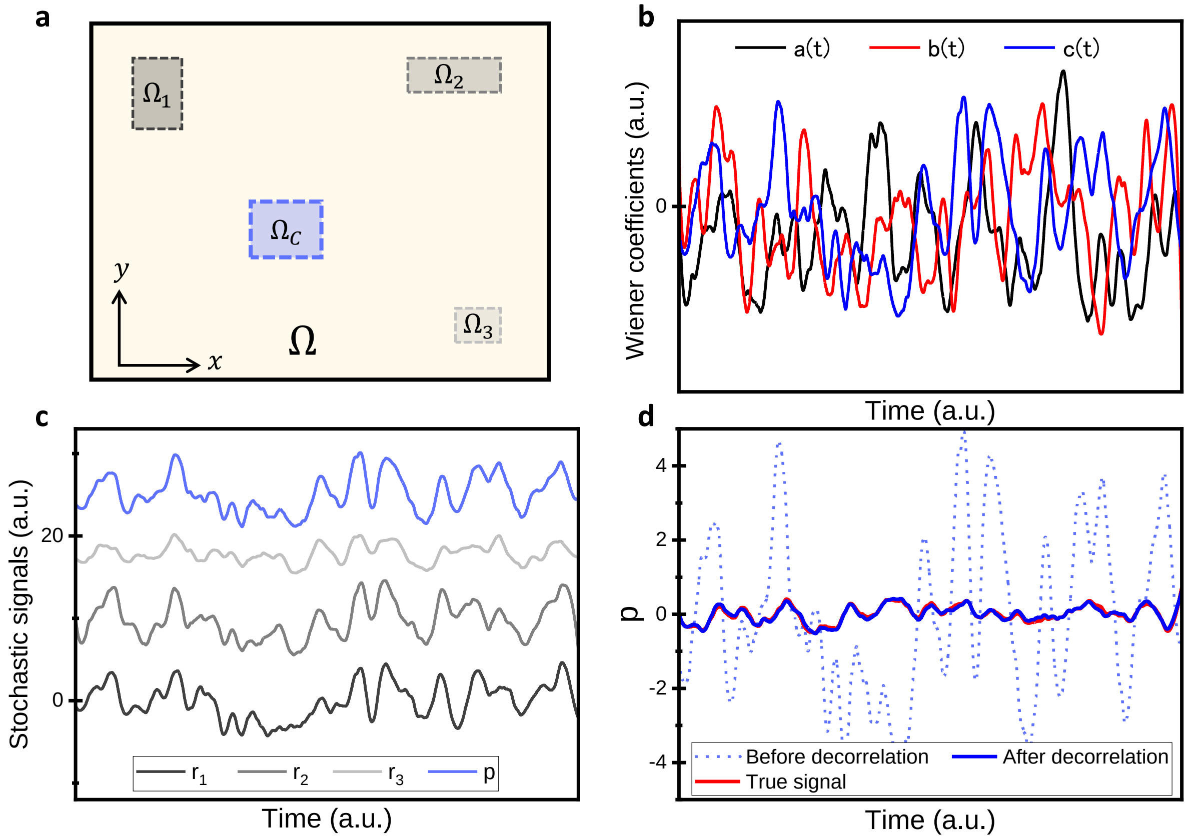

Figure S1. Numerical simulation of the effect of the noise decorrelation method, related to Figures 1 and 2. a, Representation of the 2D spatial domain of the stochastic signal $s(x, y, t)$ referred to as Ω. The signal comprises spatially correlated noise in the total domain Ω, and the stochastic signal of interest that is only generated in the subdomain Ω_C. **b,** Stochastic coefficients of the spatially correlated noise as described in Eq. (S8). **c,** Stochastic signal $s(x, y, t)$ averaged over the domains Ω_1, Ω_2, $\Omega_3$and Ω_C, referred to as r_1, r_2 , r_3 and p , respectively. The offset of each signal has been adjusted to facilitate comparison. However, the averages of the signals are zero **d,** Stochastic signal $p(t)$ before and after application of the decorrelation technique. The plot also shows the true signal without the influence of the spatially-correlated noise.

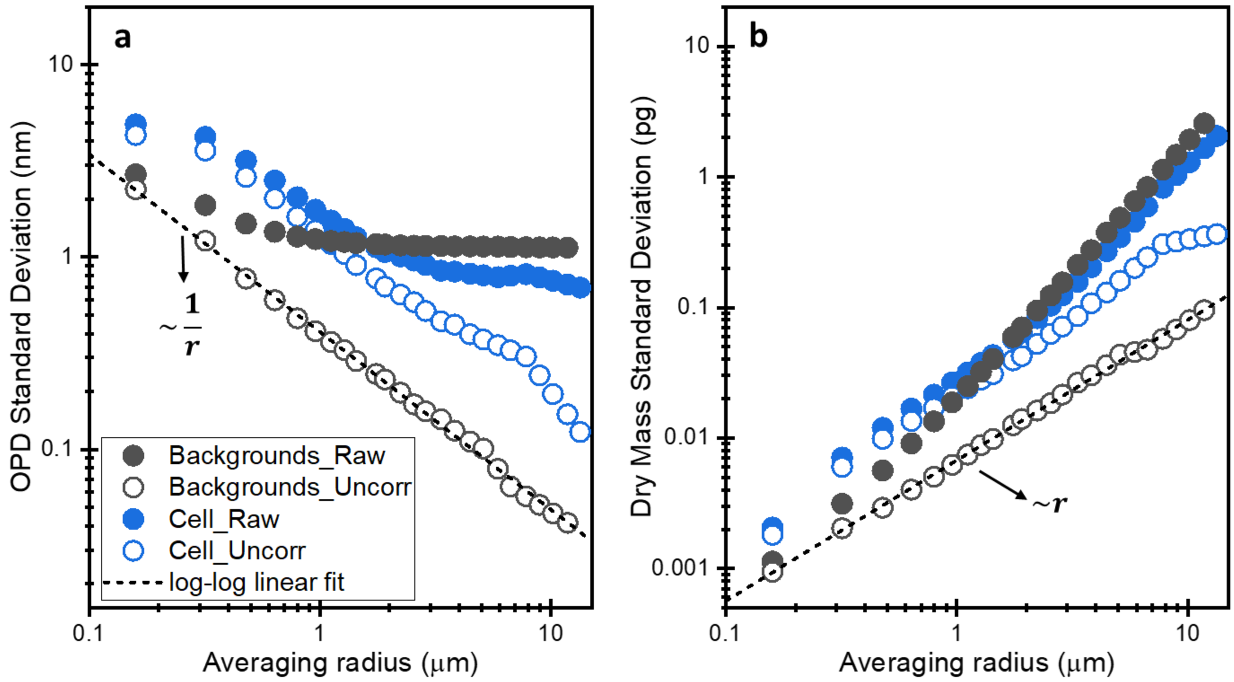

Figure S2. Effect of the noise decorrelation method on the OPD standard deviation, related to Figure 2. a, Standard deviation of the OPD for the background regions and the MCF-7 cell (as shown in Fig. 2 of the main text). The OPD standard deviations were measured before (*'raw'*) and after (*'uncorr'*) application of the decorrelation algorithm, and are plotted as a function as a function of the radius of the averaging area (r). After applying the decorrelation algorithm, the standard deviation of the OPD in the background regions shows an approximate decrease as $1/r$, indicative of white noise. **b,** Calculation of the dry mass noise derived from the background regions, along with the standard deviation of the cell dry mass fluctuations, both before and after the application of the decorrelation algorithm. The symbol labels correspond to those used in panel **a**. Given that the dry mass is directly proportional to both the OPD and the averaging area, it follows that the noise associated with the dry mass is proportional to the radius of the averaging area.

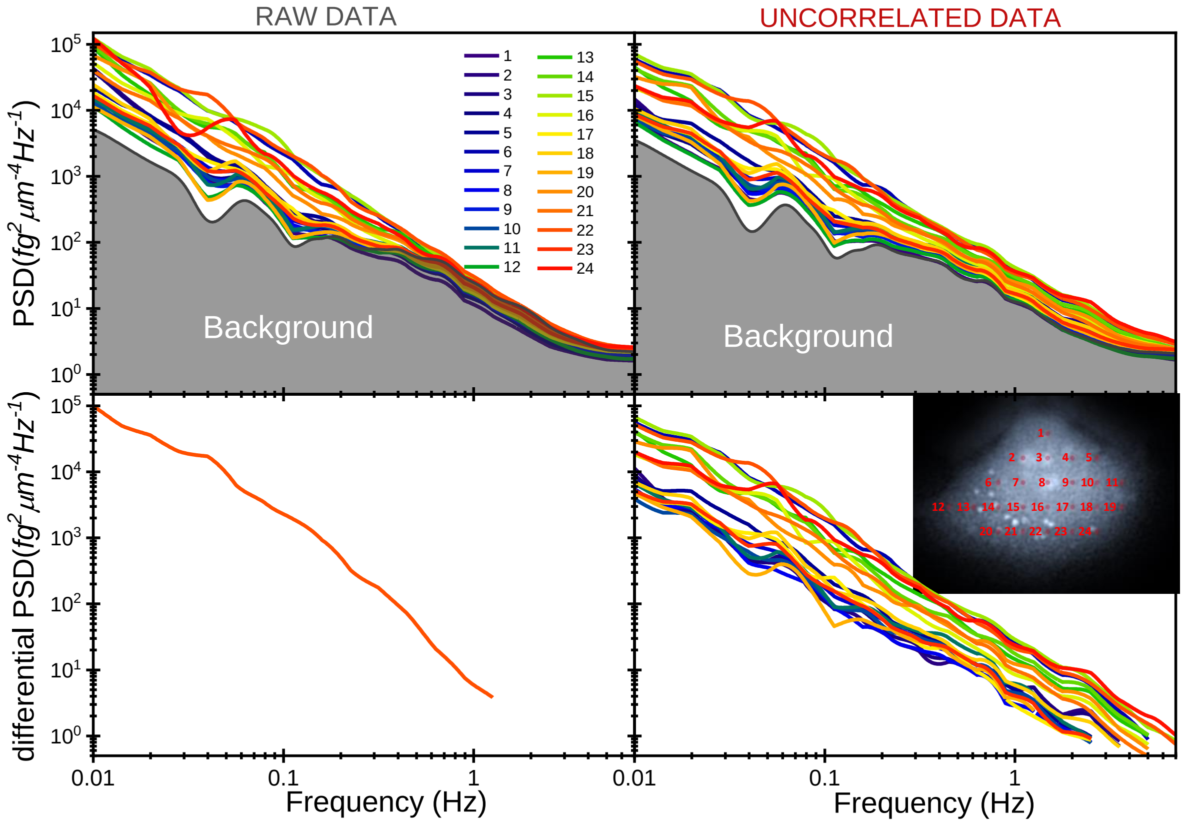

Figure S3. Effect of the noise decorrelation method on the cell PSDs, related to Figures 2 and 3. PSDs of a pixel grid within the MCF-7 cell depicted in Fig. 3 of the main text. The inset shows the grid of points on the cell. The top panels display the PSDs of the cell pixels both before and after the decorrelation algorithm is applied. The average PSDs in the background regions are also included. The decorrelation technique enhances the distinction between the cell PSDs and the background PSD, especially at higher frequencies. The selection criteria for further analysis of cell pixel PSDs require the amplitude to exceed 25% of the PSD floor in at least 70% of the frequencies. The bottom panels illustrate the differential PSDs of the cell pixels relative to the background, both before and after the decorrelation algorithm is applied. Prior to the application of the decorrelation algorithm, our selection criteria yield only one valid differential PSD out of 24, approximately 4%. In contrast, the decorrelation technique allows for the calculation of the differential PSD in 80-90% of the cell pixels.

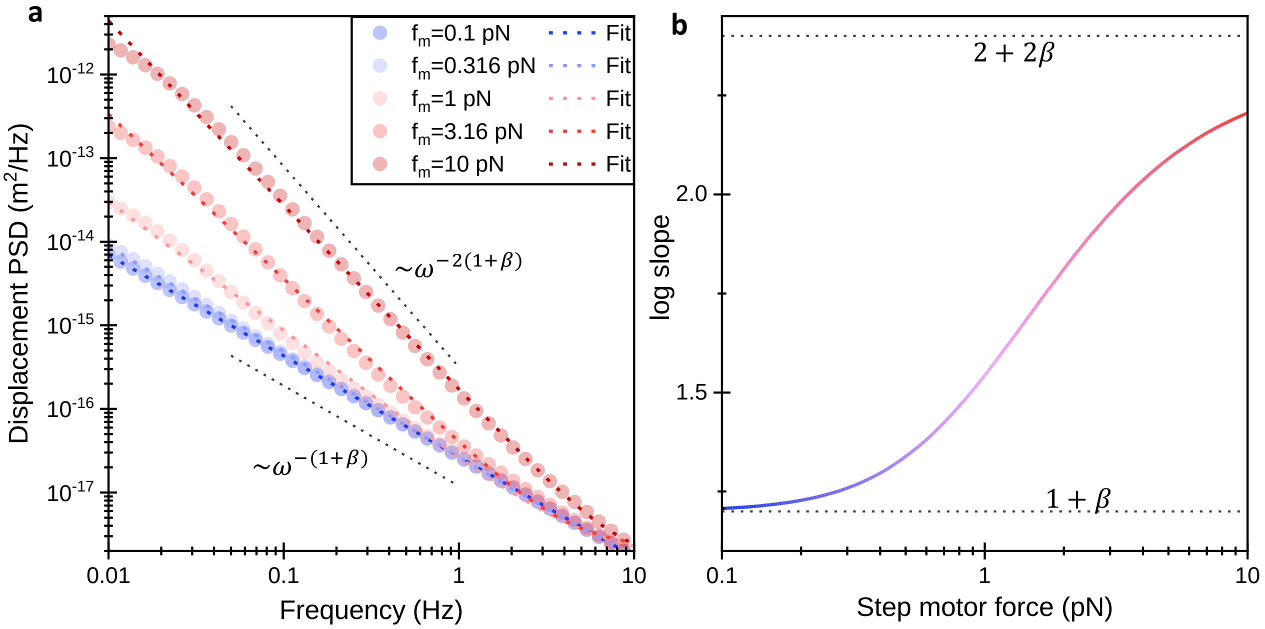

Figure S4. Theoretical simulation of the stochastic fluctuations of proteins within cells, related to Figures 3 and 6. a, Power spectral density of the stochastic displacement of a protein for several active force values, f_m, ranging from 0.1 to 10 pN. The parameters used in the simulations were k_0=30 $\mu N/m$, β=0.2, τ=10 s, similar to those found in the literature (see **Supplementary Methods**). **b,** Logarithmic slope obtained by fitting the theoretical PSDs to the phenomenological model discussed in the text.
